# Selective Iliotibial Band Release for Iliotibial Band Traction Syndrome After Second-Generation Motion-Guided Bicruciate Stabilizing Total Knee Arthroplasty and Its Rationale: A Case Report With Review of Literature

**DOI:** 10.7759/cureus.23827

**Published:** 2022-04-04

**Authors:** Naga S Cheppalli, Prabhudev Prasad Purudappa, Audrey Wassef, Jeremy Becker

**Affiliations:** 1 Orthopedics, Veterans Affairs Medical Center, Albuquerque, USA; 2 Orthopedics, Veterans Affairs Medical Center, Boston, USA; 3 Orthopedic Surgery, University of New Mexico, Albuquerque, USA

**Keywords:** guided motion, bicruciate substituting, total knee arthroplasty, traction syndrome, iliotibial band

## Abstract

Iliotibial band traction syndrome (ITBTS) after total knee arthroplasty (TKA) has been well documented following first-generation guided motion bicruciate substituting (BCS) TKA. The incidence of ITBTS following second-generation BCS has been found to be rare, and surgical release of the IT band has not been reported. A 64-year old male was diagnosed with ITBTS following second-generation guided motion BCS TKA. After a three-month trial of non-surgical treatment, he underwent selective open release of the iliotibial band (ITB), which successfully relieved his symptoms. Orthopedic surgeons should keep ITBTS as a possible differential diagnosis when evaluating the lateral-sided knee pain following guided motion BCS TKA.

## Introduction

Iliotibial band traction syndrome (ITBTS) after total knee arthroplasty (TKA) is a rare cause of painful TKA. This condition has been reported secondary to guided motion bicruciate substituting (BCS) TKA (Journey I®, Smith and Nephew, Memphis, TN, USA) [1]. However, the implant underwent substantial modification in design (second generation BCS/Journey II®) in 2011 [2]. The results of surgical management of ITBTS after the second-generation BCS knee system (BCSKS) are sparse in the literature. We present a case report with the technique, outcome, and rationale for surgical treatment (selective release of the iliotibial band [ITB]) for ITBTS following the second-generation BCSKS (Journey II®, Smith and Nephew) along with a review of literature for isolated lateral pain following TKA.

## Case presentation

**Figure 1:**
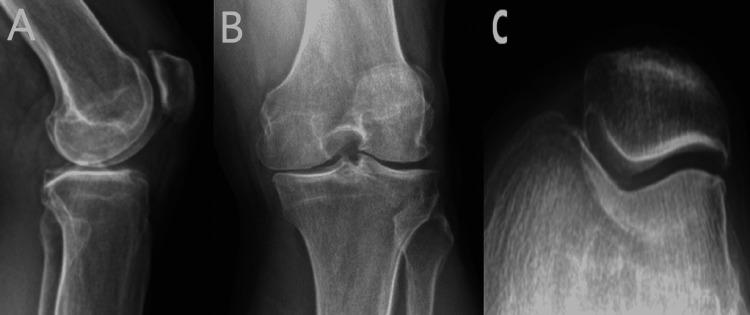
Preoperative images of left knee osteoarthritis (A) Lateral view, (B) antero-posterior view-weight bearing, and (C) skyline view

**Figure 2:**
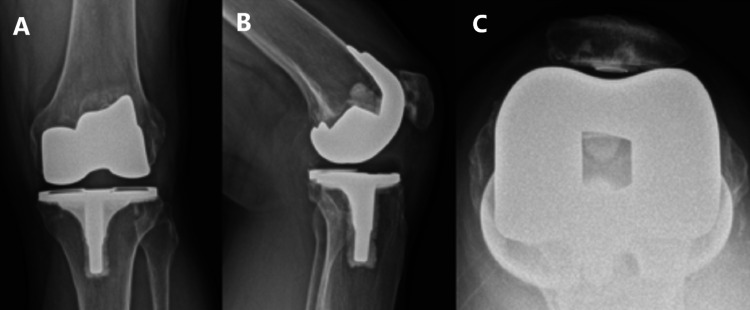
Postoperative images of left bicruciate substituting total knee arthroplasty prior to iliotibial band release (A) Antero-posterior view, (B) lateral view, and (C) skyline view

A 64-year-old male presented with end-stage medial compartment osteoarthritis (OA) of the left knee (Figure 1). After failing conservative management, the patient elected to proceed with left TKA. The patient underwent elective left TKA using the Journey II® knee system in March 2020. The standard medial parapatellar approach was utilized. The distal femur resection was done at 90° to the mechanical axis (MA) of the femur using an accelerometer (I assist®), and the proximal tibial resection was made perpendicular to the MA of the tibia using the extramedullary jig. No additional soft tissue releases other than the proximal medial tibial exposure were needed for gap balancing. The femur was measured, and a 4 in 1 cutting block was pinned at 3° external rotation to the posterior condylar axis. This step created rectangular flexion space, which was equal to extension space. Femur cuts were completed using a 4 in 1 cutting block, a box cut was performed, and tibial preparation was finished. The final implants were cemented, and 9 mm of BCS-type polyethylene was implanted. At the end of the procedure, the knee felt well balanced. Excess cement was taken out and a layered closure was obtained. The patient was discharged after 48 hours of observation. Post-operative images demonstrated a well-positioned implant without any overhang or extruded cement (Figure 2).

At the two-week follow-up visit, the patient complained of sharp pain along the lateral aspect of the knee, which persisted for three months despite NSAIDS and physical therapy. Going up and downstairs caused significant lateral-sided sharp pain. A clinical examination demonstrated no evidence of laxity with varus and valgus stress, and the patella was found to be tracking well. The patient noticed most of the pain in the mid-arc range of motion (20° to 70°). The ultrasound evaluation confirmed the fact that the point of tenderness was correlated to the anterior portion of the ITB (along with the origin of the ilio-patellar band from the ITB) at the level of the lateral epicondyle. No prominent hardware was noted, and no probe tenderness was noted at the tibial and femoral implants. A diagnostic injection with lidocaine combined with cortisone (2 ml of lidocaine and 40 mg of Kenalog) was given using ultrasound guidance at the most tender spot. This spot was located (defined using probe tenderness) along the anterior border of the ITB and the origin of the iliopatellar band (IPB) at the level of the lateral femoral epicondyle (LFEC). This procedure resulted in immediate 100% relief from the pain. The patient could climb a flight of stairs without discomfort immediately after the diagnostic block. However, the pain recurred after a week, and the patient elected to go for surgical release of the ITB with decompression of the lateral gutter.

Selective Iliotibial band release

**Figure 3:**
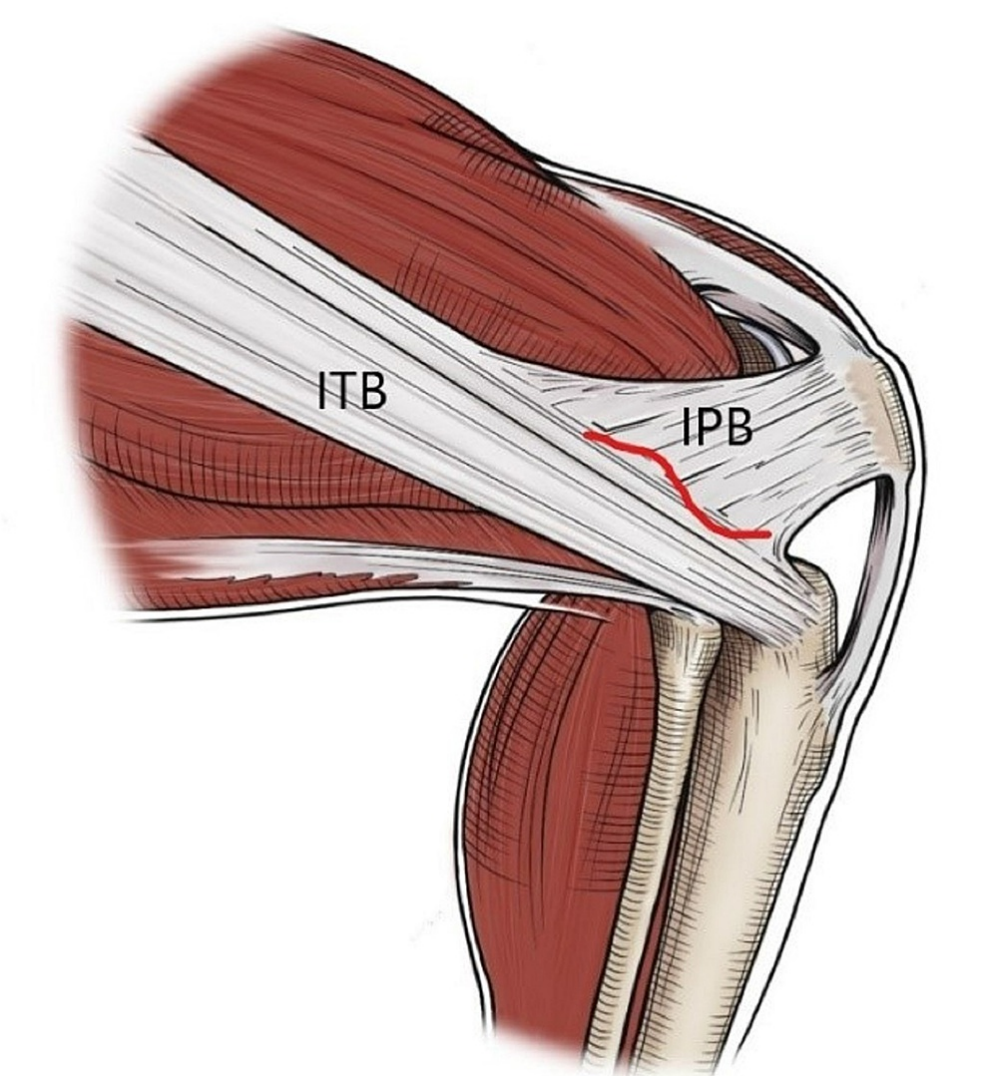
Illustration of the area of selective Iliotibial band release intraoperatively. The redline represents area of release. ITB: iliotibial band, IPB: iliopatellar band

**Figure 4:**
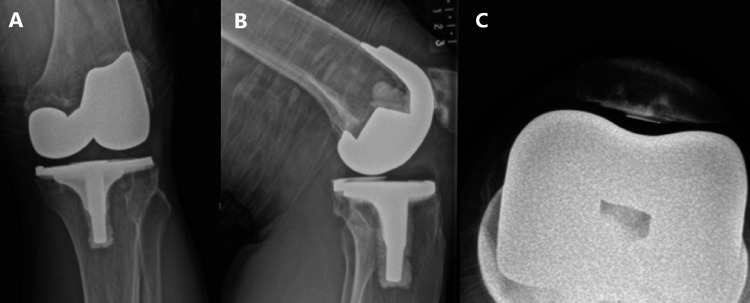
Images of left bicruciate substituting total knee arthroplasty after iliotibial band release (A) Antero-posterior view, (B) lateral view, and (C) skyline view

Under general anesthesia, with the patient in a supine position, a 3 cm longitudinal incision was placed along the anterior border of the ITB at the level of the lateral epicondyle. An IPB was identified running from the anterior border of the ITB and lateral border patella. We noted that IPB was taut when the patella was engaged in the trochlear groove. Surgical release of the IPB was performed with a lazy S-shaped incision (Figure 3). Examination of the lateral gutter demonstrated no significant osteophytes or prominent cement (Figure 4). The wound was closed in layers.

The patient's pre-operative Oxford Knee Score (OKS) was 23 (before primary TKA) and was reduced to 11 after six weeks of post-TKA and before the selective ITB release. The OKS improved significantly to 37 after the selective ITB release at a one-year follow-up.

## Discussion

Isolated iliotibial band-related pain after TKA is a very uncommon presentation. This pain could be either from the rolling of the ITB against unresected osteophytes or cementophytes, sharp edges of the implant, or implant overhang, which is collectively termed iliotibial band friction syndrome (ITBFS) [3]. However, a new clinical entity described as ITBTS has been found to be associated with motion-guided first-generation BCSKS. Luyckx et al. [1] found an incidence of 7.2% of isolated lateral knee pain with the first-generation BCS implant. ITBTS is different from ITBFS as it is secondary to the traction induced on the ITB, the iliopatellar band, and the lateral retinaculum secondary to screw home motion induced by guided motion BCSKS.

The incidence of unsatisfied TKA after surgery is around 15-20% [4]. Abnormal kinematics and sagittal instability (lack of ACL in TKA) have been reported as a cause of dissatisfaction [5]. Different concepts (kinematic alignment) and designs (BCSKS/medial pivot knee/bicruciate retaining knee) are evolving to overcome this problem. First-generation dual cam BCSKS was introduced in 2005 to reproduce normal kinematics and accounts for the lack of ACL by creating anterior cam and post [6]. This is unique from the posterior stabilized knee arthroplasty designs in many aspects (Table 1). The femoral design includes an anterior cam and a posterior cam. The anterior cam and post engage between 0° and 20° of extension, while the posterior cam engages at 60° of flexion. The asymmetrical-shaped posterior cam guides the femur into external rotation relative to the tibia during flexion (replicating the screw home motion of the native knee with intact cruciate ligaments). The BCSKS knee showed improved kinematics, better varus-valgus stability throughout the mid-flexion range, and increased stair climbing ability and ROM [7]. However, BCSKS has not shown any superior functional outcome score compared to conventional knee arthroplasty designs [8]. The BCSKS restored the A-P laxity at 15° of flexion in most knees but still has a positive pivot shift in 72% of knees, indicating the anterior cam and post have only restored the partial function of the ACL. Guided motion from the dual-cam in BCSKS induces relative internal rotation of the tibia during early flexion, coupled with the posterior translation of the lateral femoral condyle (screw home mechanism). If this motion is not synchronized, it may result in an abnormal stretch of the ITB and lateral retinaculum. Some of the recommended nonoperative treatment options for this condition are NSAIDS, physical therapy focusing on ITB stretches, and targeted corticosteroid injections. If non-surgical treatment fails, surgical release of the ITB is recommended. The first-generation BCSKS has undergone significant modifications in post position and shape, articular geometry of polyethylene, and femoral component (Journey II/Second-generation BCSKS) (Table 2). In short and midterm studies, the second-generation BCSKS knee has proven safe and effective but still reported (2%) ITBTS [3,4].

**Table 1:** Differences between designs of conventional PS and BCS knee PS: posterior stabilized, BCS: bicruciate substituting, PE: polyethylene

Conventional PS knee	BCS knee system
Posterior Cam	Dual Cam
Symmetrical PE in thickness (medial and lateral)	Asymmetrical PE shape and thickness
Neutral Joint Line	physiological joint line - 3°
Concave medial-sided polyethylene	Sagittally conforming medial side, anterior sulcus point
Concave lateral-sided polyethylene	Slight convexity on the lateral side of PE
Symmetrical medial and lateral femoral condylar thickness	Asymmetric medial and lateral femoral condylar thickness
Symmetrical and asymmetrical tibial base plate	Asymmetrical (anatomical) tibial baseplate

**Table 2:** The modifications in second-generation bicruciate substituting knee system AP: antero-posterior

Post	1. Anterior tibial post moved anteriorly	1. Allows engagement of anterior cam early during flexion
	2. Height of the post increased	2. Decreases the dislocation risk
Articular surface	1. Posterior slope increased in lateral compartment	1. Allows more flexion
	2. Posterior lip in the medial compartment moved more anteriorly	2. Decreases A-P translation in the medial compartment
Femoral component	1. Reduced thickness of medial condyle	1. Affects joint line obliquity
	2. Mediolateral width reduced	2. Avoids more prominent or overhang of the implant
	3. Lateral anterior flange reduced in thickness	3. Decreases the tension on the lateral retinaculum
	4. Posterior cam decreased in size and moved more proximally	4. Facilitates earlier engagement of posterior cam

The surgical treatment and results of selective ITB release have not been reported in terms of patient-reported outcome measures for this unique condition. We ruled out other differential diagnoses for isolated causes of lateral-sided knee pain after TKA (Table 3). Initial workup for painful TKA for infection and aseptic loosening was negative. Very localized tenderness along the anterior border of ITB and LFEC and 100% reduction of symptoms with a small amount of local anesthetic administered into the anterior portion of ITB and IPB confirmed this diagnosis. We used only 2 mL of lidocaine to prevent diffusion of the local anesthetic into the surrounding tissues. We believe this increases the accuracy of the diagnosis and helps in surgical planning. After the surgical release of tight lateral structures as described above, we noted decreased tension on the ITB and IPB during flexion of the knee joint.

**Table 3:** Differential diagnoses for isolated causes of lateral-sided knee pain after total knee arthroplasty IT: iliotibial, IR: internal rotation, ER: external rotation

Differential diagnosis	Presentation	Treatment
Popliteus impingement [9]	Snapping sensation, with pain in posterolateral and Varus stress applied to knee flexed at 90° causes pain	Surgical excision at the level of the femoral attachment
Biceps femoris tendinitis [10]	Localized tenderness along the tendon and resisted IR, and ER increases the pain	Ultrasound-guided Steroid Injection
Fabella snapping [11]	Posterolateral pain in the knee with radiological changes of fabella	Surgical excision
Overhang of tibial tray [12]	Tenderness along with IT band at the level of prominent tibial tray	Localized IT band excision
Prominent lateral border of femur	Tenderness along with prominent femoral component	Localized IT band excision
Unresected osteophytes/cementophytes [3]	Localized tenderness along with the prominence	Resection of the osteophytes and decompression of the lateral gutter
Synovitis in the lateral gutter [13]	Localized tenderness along the lateral gutter	Arthroscopic debridement

Even though the modifications in second-generation BCSKS have significantly decreased the incidence of ITBTS, this unique complication can still occur with motion-guiding second-generation BCSKS. We caution surgeons to specifically look for this problem to evaluate its real incidence. Several anatomical variations and thicknesses of ITB have been described in the literature. Future research should be aimed at evaluating if any anatomical variant of ITB is associated with an increased risk of ITBTS with guided motion BCSKS. Ultrasound significantly increases the diagnostic accuracy, and ultrasound-guided injections with local anesthesia (with or without steroids) are confirmatory and guide the surgical procedure. The surgical procedure (ITB release) is relatively benign without residual instability.

## Conclusions

This is an isolated case report of ITBTS following second-generation BCSKS and successfully treated with limited surgical release of the anterior portion of the ITB and IPB with decompression of the lateral gutter. This procedure resulted in immediate symptom relief, no instability was noted, and no further complications were reported after 18 months of follow-up.
